# Sektorenübergreifende Therapiekonzepte und innovative Technologien: neue Möglichkeiten für die Versorgung von Patienten mit psychischen Erkrankungen

**DOI:** 10.1007/s00115-021-01086-0

**Published:** 2021-03-05

**Authors:** Dusan Hirjak, Ulrich Reininghaus, Urs Braun, Markus Sack, Heike Tost, Andreas Meyer-Lindenberg

**Affiliations:** 1 Klinik für Psychiatrie und Psychotherapie, Zentralinstitut für Seelische Gesundheit, Medizinische Fakultät Mannheim, Universität Heidelberg, J5, 68159 Mannheim, Deutschland; 2 Abteilung Public Mental Health, Zentralinstitut für Seelische Gesundheit, Medizinische Fakultät Mannheim, Universität Heidelberg, Mannheim, Deutschland; 3 ESRC Centre for Society and Mental Health, King’s College London, London, Großbritannien; 4 Centre for Epidemiology and Public Health, Health Service and Population Research Department, Institute of Psychiatry, Psychology & Neuroscience, King’s College London, London, Großbritannien; 5 Abteilung Neuroimaging, Zentralinstitut für Seelische Gesundheit, Medizinische Fakultät Mannheim, Universität Heidelberg, Mannheim, Deutschland

**Keywords:** Versorgungskonzepte, Technologie, Track-Konzept, Soteria, Big Data, Therapeutic concepts, Technology, Track unit, Soteria, Big data

## Abstract

Psychische Erkrankungen sind weit verbreitet und ein bedeutendes Problem des allgemeinen Gesundheitswesens. Das Risiko, irgendwann im Laufe des Lebens eine psychische Erkrankung zu entwickeln, liegt bei rund 40 %. Psychische Erkrankungen zählen damit zu den epidemiologisch bedeutsamsten Erkrankungen. Trotz der Einführung neuerer Psychopharmaka, störungsspezifischer Psychotherapie und Stimulationstechniken zeigen viele der Betroffenen immer noch eine unzureichende Symptomremission und einen chronischen Verlauf. Durch den konzeptuellen und technischen Fortschritt der letzten Jahre wird eine neue, flexiblere und personalisierte Form der fachpsychiatrischen Patientenversorgung ermöglicht. Sowohl die traditionellen Therapie- und Organisationskonzepte als auch neuere dezentral arbeitende, modular aufgebaute, stationär-teilstationär-ambulante Einheiten werden zusammen mit innovativen digitalen Technologien vielen betroffenen Menschen mit psychischen Erkrankungen individualisierte Therapieoptionen bieten, welche ihre Symptome bestmöglich lindern und ihre Lebensqualität erheblich verbessern könnten. Das primäre Ziel der engen Verknüpfung von modernen Versorgungskonzepten und innovativen Technologien ist es, ein umfassendes Therapie- und Nachsorgekonzept (innerhalb und außerhalb der Klinik) für die individuellen Bedürfnisse von Menschen mit psychischer Erkrankung bereitzustellen. Nicht zuletzt wird dadurch auch eine ortsunabhängige Verfügbarkeit der fachärztlichen Behandlung erreicht. In der Psychiatrie des 21. Jahrhunderts müssen moderne Versorgungsstrukturen mit der aktuellen Dynamik der digitalen Transformation effektiv verknüpft werden. Die vorliegende selektive Übersichtsarbeit widmet sich den theoretischen und praktischen Gesichtspunkten eines sektorenübergreifenden Behandlungssystems kombiniert mit innovativen digitalen Technologien im psychiatrisch-psychotherapeutischen Fachbereich am Beispiel des Zentralinstituts für Seelische Gesundheit in Mannheim.

## Abstract (en)

Mental disorders are widespread and a major public health problem. The risk of developing a mental disorder at some point in life is around 40%. Therefore, mental disorders are among the most common diseases. Despite the introduction of newer psychotropic drugs, disorder-specific psychotherapy and stimulation techniques, many of those affected still show insufficient symptom remission and a chronic course of the disorder. Conceptual and technological progress in recent years has enabled a new, more flexible and personalized form of mental health care. Both the traditional therapeutic concepts and newer decentralized, modularly structured, track units, together with innovative digital technologies, will offer individualized therapeutic options in order to alleviate symptoms and improve quality of life of patients with mental illnesses. The primary goal of closely combining inpatient care concepts with innovative technologies is to provide comprehensive therapy and aftercare concepts for all individual needs of patients with mental disorders. Last but not least, this also ensures that specialist psychiatric treatment is available regardless of location. In twenty-first century psychiatry, modern care structures must be effectively linked to the current dynamics of digital transformation. This narrative review is dedicated to the theoretical and practical aspects of a cross-sectoral treatment system combined with innovative digital technologies in the psychiatric-psychotherapeutic field. The authors aim to illuminate these therapy modalities using the example of the Central Institute of Mental Health in Mannheim.

## Hintergrund

Die fachpsychiatrische und psychotherapeutische Behandlung psychischer Erkrankungen in Deutschland ist aufgrund der alternden Gesellschaft, der Zunahme der Anzahl chronisch psychisch Kranker, dem steigenden finanziellen Druck auf die Gesundheitssysteme, dem Fachkräftemangel, der Stigmatisierung psychischer Erkrankungen sowie der Unterversorgung in strukturschwachen Regionen eine Herausforderung für Therapeuten^1^ und Institutionen [1–3]. Diese Umstände führen oft zur zeitlichen Verzögerung der leitliniengerechten Therapien, negativen Folgen für den Heilverlauf mit Gefahr der Chronifizierung der Symptome und möglicherweise später eintretenden psychosozialen Nachteilen. In der klinischen Praxis erschweren lange Wartezeiten bei Fachärzten und Psychotherapeuten eine adäquate, suffiziente und evidenzbasierte Therapie mit dem Ziel der Symptomfreiheit [4]. Im Durchschnitt warten Menschen in Deutschland 12,5 Wochen auf ein Erstgespräch (s. BPtK-Studie zu Wartezeiten in der ambulanten psychotherapeutischen Versorgung, 2011; oder Barmer Arztreport 2020). Schleppende Versorgungsabläufe erzeugen Frustration bei den Betroffenen und führen oft zu Therapieabbrüchen, welche häufig zur Chronifizierung der Symptomatik führen. Aus ökonomischer Sicht ist anzunehmen, dass die benötigten Ressourcen bzw. Krankheitskosten im Bereich der psychischen Erkrankungen in Deutschland in den nächsten zwei Jahrzehnten deutlich größer werden. Die o. g. steigenden Anforderungen an die psychiatrischen Gesundheitssysteme erfordern einen Wandel in der Organisation und im Management der einzelnen Versorgungsstrukturen.

Auf der einen Seite ist eine Verbesserung der oben beschriebenen Situation durch engere Kooperation, Informationsaustausch und mögliche Abstufung verschiedener Versorgungselemente und Leistungserbringer zu erreichen. Dies schließt Haus- und Fachärzte, Psychotherapeuten und verschiedene Kliniktypen etc. mit ein. Auf der anderen Seite sollten auch verbessertes Störungs- und Behandlungswissen, neue Versorgungsmodelle und neue technische Möglichkeiten miteinander verknüpft werden, um durch optimale Ressourcennutzung die flächendeckende Versorgung psychisch Kranker in Deutschland zu verbessern:

1. *Evidenzbasierte Medizin:* Der Aufschwung der wissenschaftlichen Evidenz im Bereich der Diagnostik, Psychopharmakologie und Psychotherapie konnte zur Etablierung einheitlicher diagnostischer und therapeutischer Algorithmen führen, die derzeit in Form von S3-Leitlinien für die meisten psychischen Störungen zur Verfügung stehen.
2. *Neue Versorgungsmodelle:* In den letzten Jahren wurde eine Vielfalt moderner Behandlungssettings etabliert. Dabei spielen insbesondere die Auflösung klassischer Sektorengrenzen, die Etablierung moderner klinikinterner und -externer Versorgungsstrukturen [5, 6], die Entwicklung neuartiger Früherkennungsstrategien bzw. Prädiktionsmodelle [7], ethische Überlegungen [8] und die Bewertung der Langzeitergebnisse (therapeutisches Outcome) eine wesentliche Rolle. Zu den sektorenübergreifenden Therapiekonzepten gehören vor allem das Track-Konzept (inklusive der stationsäquivalente Behandlung [StäB]^2^; [5, 9–11]) und die Soteria [12]. Gerade in Kombination mit innovativen Technologien können die o. g. Konzepte eine qualitativ hochwertige, individualisierte und kontinuierliche Patientenversorgung leisten.
3. *Verfügbarkeit von Smartphones:* Der Anteil der Smartphone-Nutzer in Deutschland lag im Jahr 2020 bei 86 % (https://de.statista.com/statistik/daten/studie/585883/umfrage/anteil-der-smartphone-nutzer-in-deutschland; [13–15]). Ende 2020 wird es schätzungsweise 6,1 Milliarden Smartphone-Abonnements weltweit geben. Für 2026 wird ein Anstieg auf 7,5 Milliarden Nutzer weltweit prognostiziert [13].
4. *„Ambulatory assessments“:* Der Einsatz digitaler Anwendungen und Lösungen ist in unserem täglichen Leben zunehmend präsent und bietet Möglichkeiten, einige der Herausforderungen an fachpsychiatrische und psychotherapeutische Versorgung zu bewältigen [11, 12].
5. *Vernetzung von Informationen:* Das Aufkommen digitaler Technologien, einschließlich der Smartphones und tragbaren Sensoren („wearables“) zur Symptomerfassung in Echtzeit [16–20], ambulatorischer Interventionen („ecological momentary interventions“, EMI) mittels mobiler Smartphone-Applikationen [18], künstlicher Intelligenz (KI; [21]), großer Mengen multimodaler Daten (Big Data; [22]), mehrerer Technologieplattformen (computerbasierte Therapie, internetbasierte video- oder chatbasierte Behandlung, sog. „serious games“ und Virtual-Reality-Therapie; [23]), wird in den kommenden Jahren zu einer dynamischen Veränderung der klassischen Therapeut-Patienten-Beziehung führen. Sie werden nicht nur wichtige Einblicke in die Art der Umweltauswirkungen auf psychische Gesundheit liefern, sondern auch den Zugang zu psychiatrisch-psychotherapeutischen Angeboten und die Behandlungsadhärenz verbessern. Klinisch tätige Psychiater, Psychologen, Pflegekräfte, Patienten, Wissenschaftler, Politiker, Kostenträger und die Öffentlichkeit erwarten, dass digitale Technologien zur Verbesserung der Effektivität und Steigerung der Verfügbarkeit der Versorgung von Patienten mit psychischen Erkrankungen [12], zur Vermeidung unerwünschter Arzneimittelwirkungen und Reduktion der Kosten beitragen werden [24].

Grundsätzlich gilt anzunehmen, dass niederschwellige und auf soziale Kontexte und Bedürfnisse der einzelnen Patienten zugeschnittene Technologien im Sinne ambulanter Interventionen zu einer intensiveren Patientenbetreuung zur richtigen Zeit und am richtigen Ort beitragen werden [11, 12]. Blickt man auf die jüngere Literatur im angelsächsischen Raum, so haben in den letzten 10 Jahren fruchtbare ökologisch-basierte Kausalmodelle psychischer Erkrankungen und therapierelevante Konzepte den Eingang in die Versorgung von Patienten mit psychischen Erkrankungen gefunden [17–19]. Von besonderem Interesse sind dabei Prävention, Diagnosestellung und Behandlung:

1. *Sekundärprävention.* Das subjektive Erleben und die psychopathologischen Symptome der Patienten können mittels kontinuierlicher begleitender Erhebung mittels Smartphones und anderen Mobilgeräten engmaschiger erfasst werden, als es aktuell im Rahmen regelmäßiger Ambulanzkontakte möglich ist. Anstatt einer Momentaufnahme mittels klinischer Skalen ist eine kontinuierliche Messung psychopathologischer Symptome in Echtzeit und im Alltag der Patienten möglich. So werden Ärzte und Psychologen die modernen Technologien nutzen können, um aus der Ferne quantitative und objektivere Daten zu sammeln und über den Zustand ihrer Patienten informiert zu werden, um bspw. notwendige Anpassungen in der Therapie vorzunehmen, damit die Patienten so lange wie möglich in ihrer eigenen Umgebung bleiben können. Die drohende Exazerbation einer psychischen Erkrankung kann rechtzeitig erkannt und Rezidive können verhindert werden.
2. *Diagnosestellung.* Sowohl genetische Komponenten (z. B. das Zusammenwirken risikobehafteter Einzelnukleotidpolymorphismen [„single nucleotide polymorphism“, SNP]; [25] oder Genexpressionsmuster) als auch epigenetische Mechanismen (z. B. DNA-Methylierung [26], Histon- und MicroRNA-Expression etc.), welche durch Umweltfaktoren beeinflusst werden können, tragen zur Krankheitsentwicklung bei [27, 28]. Biologische Datenbanken (inkl. genetischer und epigenetischer Daten), moderne multimodale Bildgebung, mobile Datenerhebung im Alltag, kontinuierliches Monitoring der psychopathologischen Symptome und die sog. Big-Data-Analysen könnten schon bald die frühzeitige und dimensionale Diagnosestellung (inkl. neurobiologisch plausibler Diagnosegruppen), die individuelle Auswahl der Behandlungsmodalität und die Dosisanpassung der Medikation ermöglichen. Auf diesem Weg kann die psychische Gesundheit des Patienten in ihrer Ganzheit und Vielfalt erfasst und für seine Genesung genutzt werden.
3. *Behandlung.* Der Einsatz moderner Technologien in Kombination mit neuen Versorgungsmodellen wird dazu beitragen, die Wirkung und Nebenwirkungen der einzelnen Therapien zu überwachen und bei Bedarf zu optimieren. Darüber hinaus können mithilfe digitaler Technologien die Wirkfaktoren der Psychotherapie im klinischen Alltag besser erfasst und untersucht werden. Sicherlich müssen dabei die Informationssicherheit und die Privatsphäre der Patienten beachtet werden. Insgesamt sollten die digitalen Technologien die therapeutische Effizienz eines psychiatrischen Versorgungssystems verbessern, das Engagement sowie die Selbstständigkeit und Selbstbestimmungsfähigkeit („empowerment“; [17]) der Patienten fördern und die Zufriedenheit von Patienten und Klinikern steigern. Gerade während der aktuellen COVID19-Pandemie, den angeordneten sozialen Distanzierungsmaßnahmen und der zunehmenden sozialen Isolation von Menschen mit psychischen Erkrankungen [29] konnten ambulatorische Interventionen die Erbringung fachpsychiatrischer und psychotherapeutischer Behandlung im stationären (inkl. stationsäquivalenter Behandlung) und ambulanten Bereich erleichtern [30]. Beispielsweise sind viele Behandler zu Telefon- und Videosprechstunden übergegangen, um einen guten therapeutischen Kontakt trotz der notwendigen Distanzierung zu gewährleisten.

In ihrer Summe kündigen diese Entwicklungen eine neue und spannende Ära in der Psychiatrie an, die, wenn sie innerhalb einzelner Versorgungsstrukturen gefördert wird, zu wichtigen Verbesserungen bei der Versorgung von Patienten mit psychischen Erkrankungen führen kann. Sie verspricht, positive Auswirkungen sowohl für klinisch tätige Ärzte als auch für Patienten zu haben. Es ist eine Ära, in der die Kliniker und Patienten die neuen Versorgungsmodelle in Kombination mit digitaler Technologie und Datenanalysen nutzen, um in akuten Situationen (z. B. über die psychiatrische Notfallambulanz) rund um die Uhr miteinander in Kontakt treten zu können.

### Ziele der Arbeit

Die vorliegende Arbeit basiert auf selektiver Literaturrecherche und ist wie folgt strukturiert: Zuerst diskutieren wir zwei sektorenübergreifende Versorgungsstrukturen vor dem Hintergrund der aktuellen wissenschaftlichen Evidenz und liefern praktische Hinweise aus Sicht der Kliniker; dann überprüfen wir die mögliche Verknüpfung von innovativen digitalen Technologien mit den einzelnen klinikinternen und -externen Versorgungskonzepten (zu klinischen und wissenschaftlichen Zwecken), gefolgt von einer Bewertung der Grenzen der gegenwärtigen Ansätze; und schließlich machen wir uns Gedanken über weitere Pläne zur Translation von digitalen Gesundheitsanwendungen in die Patientenversorgung am Beispiel des Zentralinstituts für Seelische Gesundheit in Mannheim (ZI). Die vorliegende narrative Übersichtsarbeit zielt darauf ab, Kliniker und Forscher mit der aktuellen wissenschaftlichen Evidenz sowie den Chancen und Herausforderungen vertraut zu machen, neue Versorgungskonzepte, digitale Technologien und moderne statistische Auswertungsverfahren (z. B. KI) miteinander zu kombinieren und in die Versorgung von Menschen mit psychischen Erkrankungen zu implementieren. Diese Arbeit ist aus der Perspektive von Klinikern und Wissenschaftlern entstanden, die mit psychisch kranken Patienten arbeiten.

## Untersuchungsmethoden

Für diese Arbeit wurde bis zum 31.11.2020 eine selektive Literaturrecherche in PubMed durchgeführt, wobei die Suchbegriffe „ecological momentary assessment“, „digital technologies“, „diagnosis“, „diagnosing“, „therapy“, „psychiatric disorders“, „track concept“, „big data“, „machine learning“, „precision medicine“, „artificial intelligence“, „mental health“, und „psychiatry“ und die Bereiche, die sich mit innovativen technologischen Anwendungen in der klinischen Psychiatrie beschäftigen, verwendet wurden. Die in dieser selektiven Literaturübersicht („narratives Review“) enthaltenen Artikel wurden nicht auf systematischer Basis ausgewählt, und es wird nicht davon ausgegangen, dass die rezensierte Evidenz erschöpft ist. Die identifizierten Artikel wurden anschließend im Konsens zwischen den Autoren diskutiert.

## Ergebnisse

Die selektive Literaturrecherche unter den dargestellten Suchbegriffen konnte keine klinischen Studien zur Track-Behandlung psychischer Erkrankungen identifizieren. Zum Thema Soteria konnten insgesamt 1 kontrollierte Kohortenstudie, 1 randomisiert-kontrollierte Studie (RCT) an 2 Kohorten in den USA [31], 1 RCT in der Soteria Bern (Randomisierung durch Bettenverfügbarkeit eingeschränkt) [32–34] und 2 systematische Übersichtsarbeiten [35, 36] identifiziert werden. Nach Sichtung der Literatur lässt sich aber sagen, dass in der Soteria bis dato keine digitalen Technologien zur Diagnostik oder Behandlung eingesetzt wurden. Nicht zuletzt hat die selektive Literaturrecherche eine Vielzahl an multimodalen Studien zu innovativen statischen Modellen zur diagnostischen Abgrenzung und Vorhersage des therapeutischen Outcomes ergeben [31].

## Diskussion

Aus der aktuellen Studienlage gehen drei Hauptbefunde hervor: (1) Das Track-Konzept in der Behandlung psychischer Erkrankungen wurde bisher nicht im Rahmen klinischer Studien untersucht. (2) Soteria ist zwar ein wenig verbreitetes, aber vielversprechendes therapeutisches Konzept, wobei Untersuchungen von Wirkfaktoren der Soteria unter Einbeziehung moderner digitaler Technologien bisher nicht durchgeführt wurden. (3) Im psychiatrischen Bereich sind riesige Mengen an multimodalen Informationen verfügbar, welche mithilfe maschineller Lernverfahren für diagnostische und prognostische Zwecke genutzt werden können. In den folgenden Abschnitten werden diese Ergebnisse hinsichtlich ihrer Plausibilität und ihrer künftigen wissenschaftlichen und klinischen Anwendung diskutiert.

### ZI-Track-Konzept

Wichtige Prämissen moderner Behandlungskonzepte sind die maximale Kontinuität in einem multiprofessionellen Team (MPT) von Klinikern sowie die Vernetzung der Versorgung ohne Unterbrechung der Verantwortlichkeiten und des Informationsflusses von der akuten Aufnahme bis zur Entlassung und ambulanten Betreuung. Weitere Ziele sind die Frühintervention bei Erstmanifestationen, die Prävention von Mehrfachrezidiven und Krankheitsprogression, die Verbesserung des Wohlbefindens und der sozialen Kompetenzen bei gleichzeitiger Verringerung der Belastung der Familie sowie die Behandlung des komorbiden Substanzkonsums. Diesen Ansprüchen kann das sog. „Track-Konzept“ vollumfänglich gerecht werden [6, 9, 37]. Bisher wurde die Wirksamkeit des Track-Konzeptes nicht im Rahmen klinischer Studien wissenschaftlich untersucht. Deshalb wird an dieser Stelle ein möglicher Forschungsansatz unter Einsatz digitaler Technologien vorgestellt und diskutiert: Am ZI wird dieses Konzept Patienten mit akuten psychischen Krisen (Track-Einheit Krise und Diagnostik, KD-A) und psychotischen Störungen (Track-Einheit Schizophrenie und Psychose, SP‑A; [5]) seit mehr als drei Jahren angeboten. In der Praxis heißt es, dass Patienten in allen 5 Therapiesettings (beschützt-vollstationär, offenvollstationär, StäB, tagesklinisch und ambulant) von einem und demselben Team ohne Unterbrechung der Zuständigkeiten behandelt werden [6, 9, 37]. Digitale Technologien können die diagnostischen Maßnahmen im Track-Konzept sinnvoll ergänzen und Aussagen in Echtzeit dazu liefern, wie sich die Symptome der Patienten entwickeln. Hier fallen insbesondere zwei vielversprechende Verknüpfungspunkte auf: Zum einen können die Patienten mithilfe eines Smartphones ihr subjektives Befinden, akute Beschwerden und Symptome in regelmäßigen Abständen während des täglichen Lebens berichten. Eine solche Symptomerfassung in Echtzeit kann im Vergleich zu Symptombewertungen innerhalb einer einzigen Sitzung in einer ungewohnten Umgebung zur Steigerung der Validität beitragen [38] und ist weniger von der Fähigkeit des Einzelnen, sich über längere Zeiträume an Erlebnisse korrekt zu erinnern, abhängig [38]. Zum anderen lassen sich objektive Parameter wie z. B. Puls, Tremor, Hautleitfähigkeit, tägliche Aktivität, Sprach- und Sprechdaten sowie Mensch-Computer-Interaktion in vivo erfassen und in der nächsten Einzelsitzung mit dem Patienten therapeutisch sinnvoll nutzen. Anhand der Veränderungen der Sprachfunktion und automatisch generierten Smartphone-Daten (Bewegungsaktivität und Angaben zum subjektiven Befinden) lassen sich Zustandsmerkmale („state markers“) schizophreniformer, manischer und depressiver Episoden [39] sowie der Zeitpunkt für die Verlegung in ein anderes Setting (d. h. stationsäquivalentes, tagesklinisches oder ambulantes Setting) besser einschätzen. Darüber hinaus können diese Daten in kondensierter Form einen sog. digitalen Phänotyp liefern [14, 40]. Mithilfe des digitalen Phänotyps ergeben sich auch Chancen, erneute Exazerbationen psychotischer Störungen frühzeitig zu erkennen [41–43].

### Soteria

Der Soteria-Ansatz (nach Mosher/Ciompi; [12, 44]) ist ein stationäres Behandlungsprogramm, das darauf abzielte, den Patienten zu ermöglichen, eine psychotische Episode mit einem hohen Maß an Unterstützung und minimaler Einmischung zu bewältigen [36]. Soteria sieht eine konstante und persönliche, in erster Linie millieutherapeutische Begleitung der Patienten im Sinne einer gemeinsamen Alltagsbewältigung bzw. einer intensiven Einzelbetreuung während akuter psychotischer Krisen in einem möglichst klinikfernen Umfeld vor. Es zeichnet sich zudem durch die besondere Fokussierung auf die sozialen Interaktionen als auch den möglichst geringen Einsatz antipsychotischer (und anderer) Medikation aus [35, 45]. Die o. g. identifizierten klinischen Studien legen insgesamt nahe, dass eine Behandlung in der Soteria mindestens so effektiv (in Bezug auf Rückfälle, Symptome und Funktionsniveau) und in einigen Fällen sogar besser als die übliche Behandlung in einem psychiatrischen Krankenhaus ist [35].

**Figure 1:**
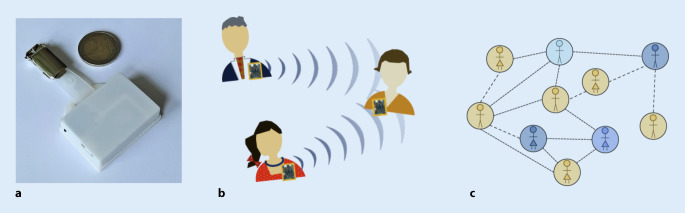

Im November 2019 startete am Zentralinstitut für Seelische Gesundheit in Mannheim das von der Klinik für Psychiatrie und Psychotherapie des Jugendalters und der Klinik für Psychiatrie und Psychotherapie interdisziplinär betriebene Adoleszentenzentrum für Psychosen – Soteria (AZP). Das AZP am ZI in Mannheim ist dabei ausgerichtet auf die Behandlung junger Menschen im Alter von 16 bis 24 Jahren, die an einer psychotischen Erkrankung oder einer Störung aus dem schizophrenen Formenkreis leiden oder die Kriterien eines Psychoserisikosyndroms (i.e. attenuiertes Psychosesyndrom gemäß DSM 5) erfüllen. Auch aufgrund der seltenen Anwendung dieses Therapiekonzeptes im deutschsprachigen Raum (Soteria lediglich an 9 Standorten in Deutschland und Schweiz verfügbar) fehlt zum aktuellen Zeitpunkt eine systematische wissenschaftliche Erforschung der zugrunde liegenden Wirkfaktoren [46]. Diesem Umstand kann u. a. durch multidisziplinäre Zusammenarbeit und innovative wissenschaftliche (digitale) Technologien begegnet werden. Darüber hinaus bieten sowohl die räumlichen und inhaltlichen Vorgaben des Soteria-Konzeptes eine einzigartige Voraussetzung, grundlegendere und für das Gesamtgebiet der Psychiatrie hochrelevante und potenziell richtungsweisende Fragestellungen zu beantworten, insbesondere die folgenden: (1) Multimodale Erhebung der komplexen sozialen Interaktionen zwischen Patienten und Mitgliedern des therapeutischen Teams (digitale Messung von räumlicher Nähe, Vokalisation und Bewegung [Gestik und Mimik], soziale Interaktion) innerhalb des milieutherapeutischen Settings in Echtzeit innerhalb der AZP-Räumlichkeiten; (2) Untersuchung des Einflusses sozialer Interaktionen auf psychotische Symptomatik als wesentlicher Bestandteil des therapeutischen Wirkmechanismus (Wirkfaktor therapeutische Beziehung nach Grawe); (3) Erforschung und objektivierte Messung psychischer und körperlicher Symptome während der Reduktions- und Absetzphase von antipsychotischer Medikation; und (4) Erforschung pathophysiologischer Krankheitsmechanismen psychotischer Erkrankungen, Störungen aus dem schizophrenen Formenkreis und Psychoserisikosyndromen in Abwesenheit medikamentöser Maskierungseffekte. Mithilfe passiver Sensortechnologie (und Kombination mit Bildgebungsmethoden) ist es inzwischen möglich, soziale Interaktionen und somit die Grundsätze der Soteria (u. a. ein unvoreingenommener Zugang zu psychotischen Erfahrungen sowie ein persönliches und gleichberechtigtes Beziehungsangebot [Mit-Sein, Mit-Tun]; [47]) zu erforschen (s. Abb. 1). Eine Klasse der derzeit eingesetzten Sensoren, sog. aktive Radiofrequenzidentifikations(RFID)-Plaketten, ermöglichen kontaktlos die relativen Distanzen zwischen Teilnehmern zu erfassen. RFIDs haben dabei den Vorteil, dass die Sensoren relativ klein und somit wenig invasiv sind, die Aufzeichnung sich auf die relative Distanzen zueinander beschränkt und die Messung für die Teilnehmer ungefährlich ist [48]. Die dabei gewonnen Daten können mittels moderner sozialer Netzwerkanalysemethoden und stochastischer Verfahren modelliert werden. Beruhend auf dem „Influence“-Model von Pentland et al. [49] wird dabei z. B. der Zustand eines Interaktionspartners von seinen interagierenden Nachbarn beeinflusst und verändert sich entsprechend. Dieser Mechanismus der zwischenmenschlichen Beziehungen bietet die Möglichkeit, den Einfluss sozialer Interaktion innerhalb therapeutischer Settings (z. B. Soteria) auf die psychopathologischen Symptome, die Kognition und das Funktionsniveau des einzelnen Patienten zu beschreiben [50].

### Präzisionsmedizin durch dimensionale störungsübergreifende Diagnostik und Big-Data-Ansatz

In der klinischen Praxis steigt seit einigen Jahren das Bedürfnis nach einer möglichst objektiven Charakterisierung von Patienten mit psychischen Störungen, etwa durch reliable neurobiologische oder neurophysiologische Marker. Traditionelle Krankheitskategorien im Sinne von ICD- und DSM-Klassifikationssystemen bilden allerdings nicht die neurobiologischen Ursachen der psychischen Störungen ab [51]. Die Kombination aus neuen digitalen Technologien und multidimensionalen Datensätze hat das Potenzial die diagnostische Klassifizierung psychischer Erkrankungen zu verändern [51]. Künstliche Intelligenz [21] und Methoden wie das „Machine Learning“ (z. B. Support-Vektor-Maschinen, moderne Algorithmen für neuronale Netzwerke oder Kreuzvalidierungsverfahren) oder „Deep Learning“ ermöglichen, hochkomplexe Datensätze aus der Klinik (subjektive Symptome und messbares Verhalten), Bildgebung (strukturelle und funktionelle Parameter) und der Genetik (genetische Hochrisikovarianten und die sog. „single nucleotide polymporphisms“) zu skalierbaren und diagnostisch verwendbaren Biomarkern zusammenzufügen. Aus der aktuellen wissenschaftlichen Evidenz wird deutlich, dass die Kombination dieser Analysetechniken mit einer Fülle multimodaler Daten aus Konsortien und Repositorien das Potenzial hat, die psychiatrischen Erkrankungen nach genetischen und hirnassoziierten Parametern sowie behavioralen Dimensionen neu zu definieren und zu diagnostizieren [51]. Ein weiteres Ziel, maschinelles Lernen und den sog. „Big-Data“-Ansatz in der klinischen Routine zu nutzen, ist die Möglichkeit, klinische Vorhersagen auf individueller Ebene zu erzielen. Das heißt, therapeutische Entscheidungen werden mithilfe multidimensionaler Daten nach Bedarf des Patienten optimiert. In der jüngsten Vergangenheit ist es mithilfe des maschinellen Lernalgorithmus LASSO (eine Methode zur linearen Regression) gelungen, einzelne Patienten mit bipolarer Störung mit einer Genauigkeit von 71 % von gesunden Probanden zu differenzieren [52]. Andere Studien haben weniger symptombasierte Variablen verwendet (z. B. neurokognitive Daten oder Neuroimaging-Scans; [53, 54]) und eine Genauigkeit von 94 % bei Identifikation und individualisierter Vorhersage klinischer Phänotypen erzielt. In der kürzlich erschienen longitudinalen Studie von Koutsouleris et al. [55] an insgesamt 334 Patienten mit Psychoserisikosyndrom oder kürzlich aufgetretener Depression ließ sich mithilfe des maschinellen Lernmodells [56], welches klinische und biologische Daten mit den Einschätzungen der Kliniker kombinierte, die Transition in eine manifeste psychotische Erkrankung in 85,9 % der Fälle korrekt voraussagen. Darüber hinaus konnte auch die reduzierte prognostische Sensitivität der Kliniker, gemessen an einer Falsch-negativ-Rate von 38,5 %, durch das sequenzielle Prognosemodell auf 15,4 % reduziert werden [55]. Die Autoren schlussfolgerten, dass ein individualisiertes prognostisches Modell, das die künstliche und menschliche Intelligenz integriert, die personalisierte Prävention psychotischer Störungen bei Menschen mit Psychoserisikosyndromen oder kürzlich aufgetretenen Depressionen erleichtern könnte. Inzwischen sind auch individualisierte Onlinerisikorechner für die transdiagnostische Vorhersage von Psychosen in der psychiatrischen Versorgung verfügbar [57–60].

Ein systematisches Benchmarking der Vorhersagbarkeit klinischer Parameter bei einzelnen Patienten kann zur Verbesserung der klinischen Symptomatik führen und das subjektive Leiden bei vielen psychischen Erkrankungen verringern [51]. Es ist ein Paradigmenwechsel, welcher eine verbesserte Auswahl der bestehenden Therapieoptionen vorsieht (entgegen dem „Trial-and-error“-Prinzip), indem deren Wirksamkeit bei einzelnen Patienten im Sinne der Präzisionsmedizin vorhergesagt wird [51]. Moderne, auf digitalen Technologien basierende, diagnostische Ansätze sind, kurz formuliert, in der Lage, dynamische Veränderung bei psychischen Erkrankungen zu erfassen und therapeutisch nutzbar zu machen. Obwohl in den letzten Jahren vielversprechende Ergebnisse erzielt wurden, müssen wissenschaftliche, ethische und datenschutzrechtliche Hürden bewältigt werden, bevor der Big-Data-Ansatz und die sog. digitale Phänotypisierung als Werkzeuge für die psychische Gesundheit der Bevölkerung eingesetzt werden können.

### Risiken und Chancen sektorenübergreifender Therapiekonzepte und innovativer Technologien

Obwohl wir vielversprechende Befunde zu sektorenübergreifenden Therapiekonzepten und innovativen Technologien in der Versorgung von Menschen mit psychischen Störungen identifiziert haben, haben sowohl die vorliegende selektive Übersichtsarbeit als auch die o. g. sektorenübergreifenden Therapiekonzepte und die innovativen Technologien mehrere Limitationen [40, 61, 62]: (1) Für die Zwecke dieser selektiven Übersichtsarbeit konnten wir zwar zahlreiche Publikationen identifizieren, die signifikante Heterogenität zwischen den veröffentlichten Studien (z. B. Einschlusskriterien, Verwendung verschiedener digitaler Interventionen, psychometrischer und neuropsychologischer Tests, die unterschiedlichen Kontrollbedingungen und Ergebnisse) und die relativ kurzen Studiendauern (in der Regel ein bis drei Monate) erschweren den systematischen Vergleich der erzielten Ergebnisse und den Transfer in die klinische Routine. (2) Es fehlen Daten zu Langzeiteffekten und den zugrunde liegenden Prozessen und Wirkmechanismen. Aus translationaler Sicht ist deshalb eine umfassende Untersuchung der Langzeiteffekte der o. g. sektorenübergreifenden Therapiekonzepte und der innovativen Technologien erforderlich. Zukünftige Studien sollten das Repertoire an Methoden erweitern und longitudinale, randomisierte und vorzugsweise transnosologische Untersuchungen durchführen, um die Schlüsselfragen zu beantworten, ob die sektorenübergreifenden Therapiekonzepte und die innovativen Technologien im Vergleich zu bereits etablierten Versorgungsstrukturen die Behandlungsqualität aus der Nutzerperspektive, die Effektivität in Bezug auf die Genesung und die Kosteneffizienz steigern können. (3) Die Therapieadhärenz der Patienten ist außerhalb von Studienprotokollen reduziert [61]. Deshalb sollten digitale Interventionen als technologiegestützte und in die modernen sektorenübergreifenden Konzepte integrierte Dienstleistungen und nicht als eigenständige Produkte betrachtet werden [40, 61, 62]. (4) Bei der Implementierung innovativer Technologien und Therapiekonzepte in der klinischen Routine müssen der Zugang zur notwendigen Technologie, der Bildungsstand, die Sprachkenntnisse, die kulturellen Besonderheiten, sensomotorische bzw. kognitive Defizite und die Psychopathologie (Cave: Angst, Misstrauen, technischer Beeinflussungs- oder Beeinträchtigungswahn [63]) der Patienten berücksichtigt werden. Darüber hinaus sollten die Kliniker und Forscher auch auf den Bedarf an neuen Weiterbildungsmöglichkeiten für Therapeuten achten. Deshalb sollen in der Zukunft sektorenübergreifende Therapiekonzepte und innovative Technologien für die psychiatrische Versorgung in enger Zusammenarbeit mit den Patienten oder anderen relevanten Interessengruppen (z. B. Angehörige) entwickelt und evaluiert werden. (5) Die longitudinale und qualitativ hochwertige Erfassung klinischer und neurobiologischer Daten stellt nach wie vor eine große Herausforderung dar. Konzepte, wie solche Daten zentral, standardisiert und wissenschaftlich verwertbar erhoben werden, befinden sich derzeit noch in der Entwicklung. Entscheidend für eine breite Akzeptanz ist dabei nicht nur, dass der einzelne Patient unmittelbar von der Erhebung seiner Daten profitiert, sondern auch, dass diese Daten für Kliniker direkt nutzbar gemacht werde, um bereits vor der Entwicklung komplexer Modelle und Entscheidungshilfen mittels künstlicher Intelligenz eine qualitativ hochwertige und quantitative klinische Entscheidungsgrundlage bieten zu können. (6) Der Schutz, das Speichern und Aufbewahren von Patientendaten und Informationen zur psychischen Gesundheit der Betroffenen, welche mit digitalen Technologien erhoben werden, ist für Kliniker, Forscher und Patienten ein hochrelevantes Thema. Patienten, Ärzte und Forscher sollten die Nutzungsbedingungen digitaler Technologien sorgfältig prüfen, bevor sie sensible, persönliche Daten eingeben. Insbesondere sollten sie prüfen, welche Daten gesammelt werden, wer Zugriff auf diese Daten hat und wie die Privatsphäre der Beteiligten geschützt wird [40]. Dabei sind vor allem die Betroffenenrechte im Sinne der EU-Datenschutz-Grundverordnung (EU-DSGVO) zu beachten. (7) Nicht zuletzt muss die Akzeptanz der sektorenübergreifenden Versorgungsstrukturen und der innovativen Technologien bei den Kostenträgern gesteigert werden.

## Fazit für die Praxis

Die Versorgung von Menschen mit psychischen Erkrankungen hinkt bei der Implementierung eines präzisionsmedizinischen Ansatzes für Prävention, Diagnostik und Therapie hinter anderen medizinischen Bereichen hinterher. Die Kombination aus modernen Versorgungsmodalitäten, jüngsten Fortschritten in der Genetik und Bildgebung sowie innovativen digitalen Technologien wird helfen, diesen Rückstand aufzuholen und maßgeschneiderte, durch moderne Auswertungsverfahren gestützte diagnostische Maßnahmen und Therapien für Patienten jederzeit und überall verfügbar zu machen.

## Footnotes

1 Anmerkung: Im Interesse einer besseren Lesbarkeit wird in dieser Arbeit nicht ausdrücklich in geschlechtsspezifischen Personen- bzw. Patientenbezeichnungen differenziert. Die gewählte männliche Form schließt eine adäquate weibliche Form gleichberechtigt ein.

2 Digitale Versorgungsformen zur Personalisierung der StäB sollen im Rahmen einer eigenständigen selektiven Übersichtsarbeit in dieser Zeitschrift dargestellt werden.
